# Association of Variants in *CDC10* (*Septin 7*) Gene with Growth-Related Traits in Qinchuan Cattle

**DOI:** 10.3390/ani16030447

**Published:** 2026-02-01

**Authors:** Zixuan Cheng, Yongli Yue, Yilin Wang, Peinuo Zhou, Xinyue An, Jianyu Xu, Takahisa Yamada, Gong Cheng, Hugejiletu Bao, Linsen Zan, Bin Tong

**Affiliations:** 1 The State Key Laboratory of Reproductive Regulation and Breeding of Grassland Livestock, School of Life Sciences, Inner Mongolia University, Hohhot 010021, China; chengzixuan2577@163.com (Z.C.); yueyongli228@163.com (Y.Y.); wangyilin510sl@163.com (Y.W.); m2572770276@163.com (X.A.); xujianyuimu@163.com (J.X.); 2 Inner Mongolia Autonomous Region Agricultural and Animal Husbandry Technology Extension Center, Hohhot 010013, China; zhoupeinuo_zoe@163.com (P.Z.); baohuge@imnu.edu.cn (H.B.); 3 Department of Agrobiology, Faculty of Agriculture, Niigata University, Niigata 950-2181, Japan; tyamada@agr.niigata-u.ac.jp; 4 National Beef Cattle Improvement Center, College of Animal Science and Technology, Northwest A&F University, Xianyang 712100, China; chenggong@nwafu.edu.cn (G.C.); zanlinsen@163.com (L.Z.)

**Keywords:** association, *CDC10*, growth-related traits, Qinchuan cattle, SNP

## Abstract

As one of the five major indigenous yellow cattle breeds in China, Qinchuan cattle are characterized by stable genetic performance and desirable meat quality. However, compared with imported commercial breeds, Qinchuan cattle have a relatively slow growth rate. Therefore, improving the growth rate of Qinchuan cattle has become a top priority in Qinchuan cattle breeding. The *CDC10* (*Septin 7*) gene, an important member of the Septin family, participates in various cellular physiological processes including intracellular substance transport, cell division, cell cycle regulation, and apoptosis. Studies have repeatedly mapped the *CDC10* gene to quantitative trait loci influencing growth-related traits, such as body weight and carcass weight in many beef cattle breeds. Previous study has also demonstrated the high expression of *CDC10* in JB cattle with high performance for carcass weight, however, the association between *CDC10* and growth-related traits in Qinchuan cattle remain unclear. Therefore, in this study, we selected five individuals each from Chinese Simmental, Mongolian cattle, Luxi cattle, and Qinchuan cattle for direct sequencing, aiming to identify mutations within the *CDC10* gene of native Chinese yellow cattle. Subsequently, we performed genotyping of 367 Qinchuan cattle using the MassARRAY technology, followed by genetic diversity analysis of the identified mutations and association analysis between these sites and growth-related traits of Qinchuan cattle. This study demonstrated high expression of the *CDC10* gene in Qinchuan cattle with high performance for carcass weight. Furthermore, we identified the g.61303052G>C and c.225A>G SNPs in the promoter and exon regions, respectively, as being significantly associated with multiple growth-related traits in Qinchuan cattle. The c.225A>G SNP was also found to alter the secondary structure of the CDC10 protein. These findings provide reliable molecular markers for enhancing the growth rate of Qinchuan cattle and establish a solid theoretical foundation for the development of the beef cattle industry.

## Simple Summary

The growth-related traits of beef cattle are core indicators for measuring their production performance and economic value. The *CDC10* (*Septin* 7) gene is an important component of the Septin family and is involved in various cellular life activities. In recent years, the *CDC10* gene has been repeatedly mapped to quantitative trait loci (QTL) affecting growth-related traits in many cattle breeds. This study revealed that both the g.61303052G>C (ss9673029750) SNP in the promoter region and the c.225A>G (ss9673029758) SNP in exon 4 of the *CDC10* gene are significantly associated with growth-related traits in Qinchuan cattle through association analysis. This research provides effective molecular markers for improving the growth rate of Qinchuan cattle.

## 1. Introduction

The beef cattle industry is one of the fastest-growing sectors in China’s animal husbandry sector. In recent years, China has become a key player in global beef production and consumption. As one of the five major indigenous yellow cattle breeds, Qinchuan cattle are highly valued for their tender meat, adaptability to roughage, and strong environmental resilience [1]. Their unique physical characteristics and superior meat quality make them widely popular. However, compared to introduced commercial breeds, Qinchuan cattle exhibit slower growth rates and underdeveloped hindquarters [2]. For many years, production practices have shown that crossbreeding using Qinchuan cattle as the female parent with heavy-sized cattle breeds imported from abroad, or using Qinchuan cattle as the male parent with small-sized local cattle breeds from other regions, generally yields good results [3]. Nevertheless, as time goes by, this method of improving the growth-related traits of Qinchuan cattle through interbreed crossbreeding has encountered some drawbacks, such as breed confusion and heterosis degradation.

The development of molecular marker-assisted breeding technology in beef cattle has primarily gone through three stages: molecular markers based on molecular hybridization techniques, such as restriction fragment length polymorphism (RFLP); PCR-based molecular markers, including microsatellite markers and PCR-single strand conformation polymorphism (PCR-SSCP); and sequencing-based molecular markers, represented by single nucleotide polymorphism (SNP) [4,5]. Among these, SNP is widely applied in marker-assisted breeding for beef cattle due to its abundance, extensive distribution throughout the genome, high polymorphism, and suitability for large-scale, high-efficiency screening [5]. Therefore, relying on molecular marker-assisted breeding technology to increase the growth rate of Qinchuan cattle has become a top priority for the Qinchuan cattle industry.

Growth-related traits in beef cattle are core indicators for measuring production performance and economic value, jointly regulated by polygenes, multiple loci, and environmental factors [6,7]. Previous studies have identified many genes influencing the growth-related traits of Qinchuan cattle through GWAS analysis, such as *ADAMTS17*, *ALDH1A3*, *CHSY1*, *MAGEL2*, *MEF2A*, *SYNM*, *CNTNAP5*, and *CTNNA3* genes [8]. Nevertheless, the molecular and genetic regulatory mechanisms underlying growth-related traits in Qinchuan cattle have not yet been fully elucidated. This study focused on the cell division cycle 10 (*CDC10*) gene, also known as *septin 7*, which is a member of the Septin family involved in various cellular biological processes [9,10,11]. In a cattle study, the *CDC10* gene has been consistently mapped to quantitative trait loci (QTLs) associated with growth-related traits across multiple beef cattle breeds [12,13,14,15]. Our previous studies have found that the expression levels of the *CDC10* gene were higher in the skeletal muscle in Japanese Black beef cattle (JB), with high growth performance than in JB with low growth [16]. The aforementioned findings suggest that the *CDC10* gene could be considered as a strong candidate gene for growth-related traits in beef cattle. Nevertheless, the association between the *CDC10* gene and growth-related traits in Qinchuan cattle remains unclear. In this study, Western blot was used to compare the differential expression of CDC10 protein between Qinchuan cattle with high and low growth performance for carcass weight. Subsequently, five individuals each of Chinese Simmental cattle, Mongolia cattle, Luxi cattle, and Qinchuan cattle were selected for direct sequencing to identify mutation in the *CDC10* gene of indigenous Chinese yellow cattle. MassARRAY technology was then employed to genotype 367 Qinchuan cattle, followed by genetic diversity analysis and association analysis with growth-related traits. Finally, physicochemical properties and protein secondary structure were analyzed for the SNP located in the exon. This study aimed to identify mutations in the *CDC10* gene and perform an association study with growth-related traits in Qinchuan cattle breed, thereby providing reliable molecular markers for improving the growth rate of Qinchuan cattle and offering effective scientific insights for the development of the Qinchuan cattle breeding industry.

## 2. Materials and Methods

### 2.1. Animals

The DNA samples of 367 clinically healthy and non-pregnant Qinchuan adult females (aged 24 to 30 months, and unrelated for at least three generations) were provided from the National Beef Cattle Improvement Center, Northwest A&F University [17]. Animals were chosen at random from the National Beef Cattle Improvement Center herd (Xianyang, China). All cattle were fed the same total mixed ration with a roughage to concentrate ratio of 3:2. Specifically, the roughage fraction of the diet was composed primarily of corn silage (accounting for 55% of the total diet) and wheat straw (5% of the total diet), while the concentrate fraction consisted of corn (23%), soybean meal (7.5%), wheat bran (4.5%), sodium chloride (1%), ammonium hydrogen phosphate (1%), and a commercial premix (3%). This TMR formulation provided 12.08% crude protein, alongside 32.85% neutral detergent fiber, 18.71% acid detergent fiber, and 3.66% ether extract. The cattle herd was managed under standardized conditions at the National Beef Cattle Improvement Center, Northwest A&F University, which is located in a region characterized by a semi-humid and semi-arid climate with an annual average temperature of 15.1 °C. The feeding was offered based on NRC standards [18]. Body weight (BW) was regarded as an important growth trait, and body measurement traits including body length (BL), wither height (WH), hip height (HH), rump length (RL), hip width (HW), chest depth (CD), chest circumference (CCF), and pin bone width (PWB) were measured using a measuring tape, measuring stick, and sliding caliper [19]. The longissimus dorsi meat quality traits including loin muscle depth (ULD), loin muscle area (ULA), intramuscular fat (IMF), and back fat thickness (BFT) were estimated using ultrasound technology (Sono-grader ultrasound machine, Renco, Boca Raton, FL, USA) [20]. The phenotypic traits were measured through ultrasonography, because ultrasound measurement predicts meat quality traits in live animals in a nondestructive manner. The ultrasonic probe was placed in the area between the 12th and 13th ribs [21,22]. The genomic DNA of 367 Qinchuan cattle were extracted according to the standard phenol chloroform protocol [23]. The quality and quantity of the extracted DNA were evaluated using a Nanodrop^®^ spectrophotometer (ensure that the A260/A280 ratio is within the range of 1.8–2.0 and the A260/A230 ratio is greater than 2) (Thermo Fisher Scientific, Waltham, MA, USA) and by agarose gel electrophoresis (a distinct high-molecular-weight main band was observed with no obvious degradation).

### 2.2. CDC10 Sequencing and Variant Detection

**Figure 1:**
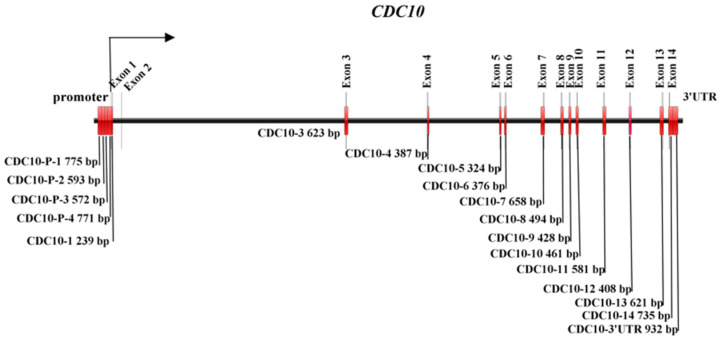
*CDC10* gene PCR primer location diagram.

Twenty cattle (including five Chinese Simmental (CS), five Qinchuan cattle, five LX cattle and five IM cattle) were used to detect variants in the *CDC10* gene. Bovine specific PCR primers were designed using Primer 5.0 software (Premier Biosoft International, Palo Alto), according to the bovine *CDC10* DNA sequence (NCBI reference sequence: NC_037331.1) (Figure 1 and Table 1). PCR amplifications were performed with 2 μL of the prepared DNA as a template in a final volume of 50 μL containing 1 μM of each primer, 25 μL of Ex Taq DNA polymerase (Takara, Kusatsu, Japan), and 21 μL of ddH_2_O. The PCR conditions were as follows: 94 °C for 5 min, 35 cycles of 94 °C for 30 s, annealing for 30 s, 72 °C for 1 min 10 s and a final extension step at 72 °C for 10 min. The annealing temperatures for each fragment are shown in Table 1. The PCR products were analyzed by 1.0% agarose gel electrophoresis to determine DNA sequencing quality and quantity. The products were sequenced by the Beijing Genomics Institute (Beijing, China).

### 2.3. SNP Genotyping by iPLEX MassARRAY

The 13 variants were genotyped with the MassARRAY^®^ SNP genotyping system (Agena Bioscience, San Diego, CA, USA) in the 367 Qinchuan populations. PCR and extension primers were designed from sequences containing each target mutation and ~100 upstream and downstream bases with Assay Design Suite (Table 2) (http://agenabio.com/assay-design-suite-20-software, accessed on 20 August 2025), using the default settings. The genotype of each SNP was analyzed using the Sequenom MassARRAY iPLEX platform (Sequenom, San Diego, CA, USA) [24]. The resulting data were analyzed using the MassARRAY Typer 4.0 Analyzer software (Agena Bioscience, San Diego, CA, USA).

### 2.4. Western Blot

To compare the differential expression of the CDC10 protein, we selected three individuals with the highest body weight and three with the lowest body weight (all six Qinchuan cattle were 28 months females) from a population of 367 Qinchuan cattle and performed using biopsy to collect samples of the *longissimus thoracis* muscle at the 12–13th ribs for subsequent analysis. The *longissimus thoracis* muscle samples from Qinchuan cattle were rapidly frozen in liquid nitrogen, pulverized into powder, and lysed in radioimmunoprecipitation assay (RIPA) buffer (Beyotime, Shanghai, China) supplemented with phenylmethylsulfonyl fluoride (PMSF). Total protein was separated by SDS-PAGE and transferred to nitrocellulose (NC) membranes (Pall Corporation, East Hills, NY, USA). Membranes were blocked with 5% skim milk for 2 h at room temperature, incubated with primary antibodies overnight at 4 °C, and then with secondary antibodies for 1 h at room temperature. Protein bands were visualized using chemiluminescent reagents (Thermo, Waltham, MA, USA). Band intensities were quantified with ImageJ (https://imagej.net/ij/, accessed on 29 September 2025). The primary antibodies used were CDC10 (1:1000, Abcam, Cambridge, UK) and α-Tubulin (1:1000, Proteintech, Rosemont, IL, USA); α-Tubulin served as the loading control. The secondary antibodies were anti-rabbit (1:10,000, Proteintech) and anti-mouse (1:10,000, Proteintech). Target proteins were detected with the Tanon-5200 imaging system (Tanon, Shanghai, China) according to the manufacturer’s instructions.

### 2.5. Quantitative Real-Time PCR

This experiment utilized the RNeasy^®^ Mini Kit (Qiagen, Düsseldorf, Germany) to isolate the total RNA from samples of the above-mentioned *longissimus thoracis* muscle, followed by cDNA synthesis using the reverse transcription kit (Takara, Kusatsu, Japan). After assessing RNA purity and concentration by spectrophotometry using a NanoDrop 2000 (Wilmington, NC, USA) and 1% agarose gel electrophoresis, quantitative real-time PCR (RT-PCR) was performed using TB Green^®^ Premix Ex Taq™ (Tli RNaseH Plus) (Takara) and specific primers (*CDC10*-F: 5′-TGTTTATACTTCATTGCTCCT-3′, *CDC10*-R: 5′-TTAAACTGTTGGCATTCCTC-3′) on a Bio-Rad system (Bio-Rad, Hercules, CA, USA). Each experimental group was repeated at least three times. The 2^−ΔΔCt^ method was employed to calculate and analyze statistically significant differences in gene expression based on Ct values. *GAPDH* was used as the internal controls.

### 2.6. Statistical Analysis

Genotypic, allelic frequencies and Hardy–Weinberg equilibria were calculated for Qinchuan breed. Population genetic indices, observed heterozygosity (H_o_), expected heterozygosity (H_e_), effective allele numbers (n_e_), and polymorphism information content (PIC) were calculated according to Nei’s method [25]. Allelic frequencies of the 13 variants were compared by an χ^2^ test. The linkage disequilibrium (LD), including D’ and *r*^2^, was assessed with HAPLOVIEW v. 4.2 (https://haploview.software.informer.com/#google_vignette, accessed on 2 October 2025) [26]. Haplotypes were obtained using SHEsis [27]. The bioinformatic tools and online resources used in this study for the sequence analysis and prediction are summarized in Table S1. The relationships between 13 traits and different genotypes in Qinchuan were analyzed in SPSS 24.0 (SPSS, Inc., Chicago, IL, USA). The statistical linear model for these analyses was consistent with our previous report [17] *Y_ij_* = *μ* + *G_i_* + *A_j_* + *e_ij_*, where *Y_ij_* means trait value per individual, *μ* means overall population mean per trait, *G_i_* means fixed effect associated with genotype, *A_j_* means fixed effect of age, and *e_ij_* represents random error. When the number of cattle with a given genotype was less than ten, their correlations and effects could not be reliably estimated. Therefore, animals with this genotype were excluded from the analysis. The Bonferroni correction was used to adjust *p* values [17]. All charts were created using GraphPad Prism 7.0 (https://www.graphpad.com/, accessed on 3 October 2025) and the data represented the mean ± SEM. The significance of differences between the groups was assessed using the Student’s *t* test (*, *p* < 0.05; **, *p* < 0.01 ***, *p* < 0.001).

## 3. Results

### 3.1. Description of the Correlations Among the 13 Growth-Related Traits in Qinchuan Cattle

**Figure 2:**
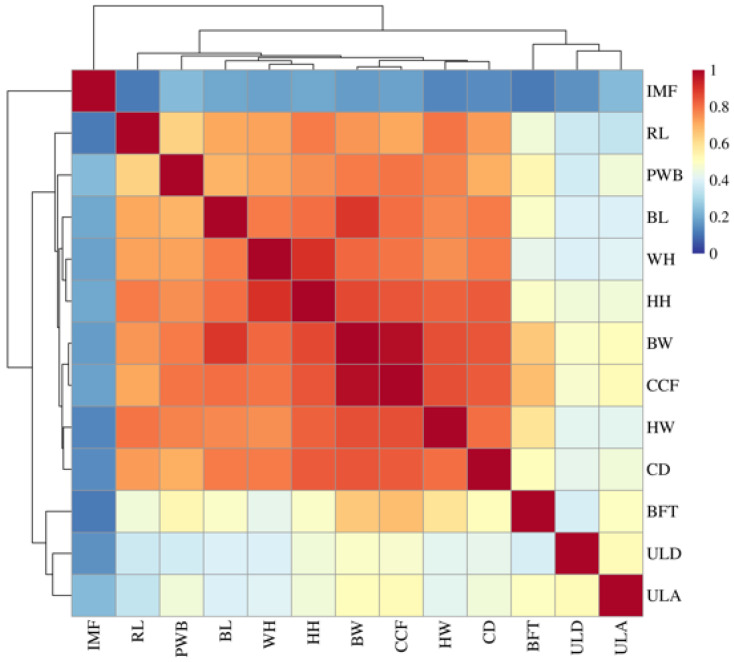
Correlations among the 13 growth-related traits in Qinchuan cattle. Strong correlations are represented in red, and weak correlations in blue. The color change from blue to red is directly proportional to the magnitude of the association coefficient. BW, Body weight; BL, Body length; WH, Wither height; HH, Hip height; RL, Rump length; HW, Hip width; CD, Chest depth; CCF, Chest circumference; PWB, Pin bone width; BFT, Ultrasound back fat thickness; ULD, Ultrasound loin muscle depth; ULA, Ultrasound loin muscle area; IMF, Ultrasound intramuscular fat.

In breeding programs, it is essential to consider the correlations among multiple traits. Therefore, this study analyzed the correlations among 13 growth-related traits of Qinchuan cattle using a heatmap. The results showed strong correlations among the growth-related traits BW, WH, HH, RL, HW, CD, CCF, and PWB, forming a distinct reddish-orange block in the heatmap (Figure 2). This suggests that these growth-related traits in Qinchuan cattle can mutually influence one another. The specific data for the 13 growth-related traits are presented in Table S2, while the association coefficients and corresponding *p*-values for each trait are provided in Table S3.

### 3.2. Differential CDC10 Expression Between High and Low Growth Performance for Carcass Weight

**Figure 3:**
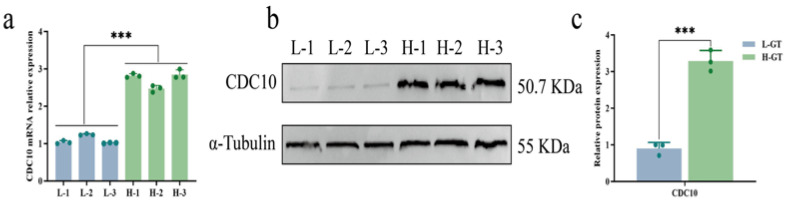
Differential CDC10 expression between high and low growth performance for carcass weight. (**a**,**b**) RT-PCR and Western blot was used to detect differences in CDC10 mRNA and protein expression between Qinchuan cattle with high (H-1, H-2, and H-3) and low (L-1, L-2, and L-3) growth performance for carcass weight. (**c**) Quantitative analysis of CDC10 protein. *** *p* < 0.001. As below.

RT-PCR and Western blot analysis revealed that CDC10 expression was significantly higher in the H-GT group than in the L-GT group (*p* < 0.001, Figure 3).

### 3.3. Detection of CDC10 Genetic Variants

**Figure 4:**
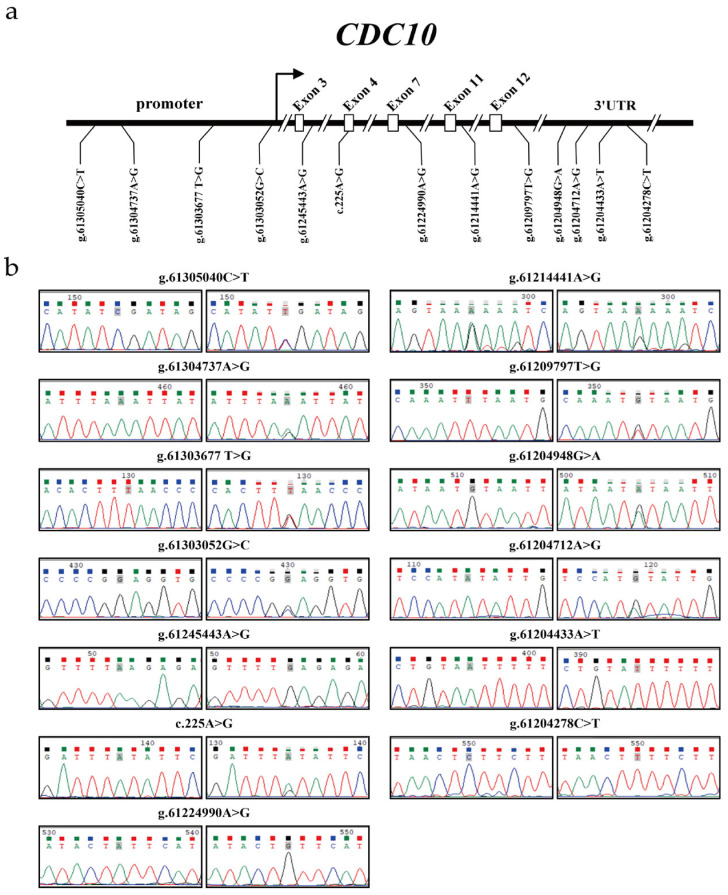
Detection of variants in bovine *CDC10*. (**a**) The physical locations of each of the 13 variants identified in this study are shown. (**b**) Nucleotide substitutions of the 13 *CDC10* variants are shown (GenBank accession: NC_037331.1).

Twenty cattle (including five CS, five Qinchuan cattle, five LX cattle and five IM cattle) were used to detect novel variants in the *CDC10* gene. The sequence analysis revealed 13 variants in the *CDC10* gene. Among them, the known g.61303052G>C (ss9673029750) variant was detected in the promoter region of *CDC10* in Chinese cattle breeds. The novel g.61304737A>G (ss9673029668), g.61305040C>T (ss967302973), and g.61303677T>G (ss9673029652) variants were found in the promoter region of *CDC10*. The novel g.61245443A>G (ss9673029723), c.225A>G (ss9673029758), g.61224990A>G (ss9673029704), g.61214441A>G (ss9673029743), and g.61209797T>G (ss9673029716) variants were found in intron 3, exon 4, intron 7, intron 11, and intron 12 of *CDC10*, respectively. Meanwhile, the g.61204948G>A (ss9673029718), g.61204712A>G (ss9673029657), g.61204433A>T (ss9673029479), and g.61204278C>T (ss9673029726) variants were found in the 3′UTR region of *CDC10* (Figure 4) (the variant data for this study have been deposited in the European Variation Archive (EVA) at EMBL-EBI under accession number PRJEB57636 (https://www.ebi.ac.uk/eva/?eva-study=PRJEB57636, on 20 September 2025).

**Figure 5:**
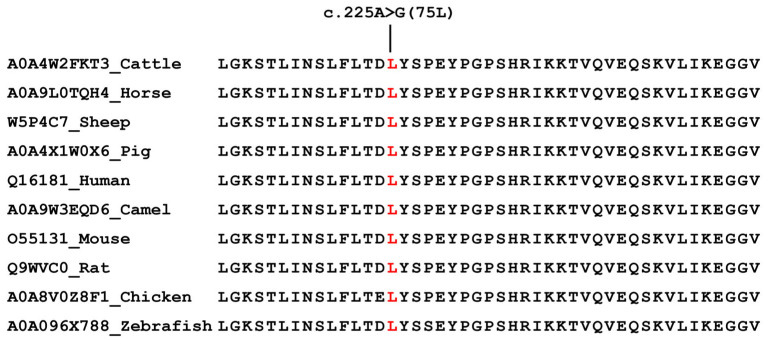
Alignment of the wild-type sequence in various species in the CDC10 protein. CDC10 multispecies alignment in the region of synonymous mutations. CDC10 amino acid sequence of each species was obtained from the UniProt database. The red “L” denotes leucine.

The c.225A>G mutation in exon 4 of CDC10 caused silent mutation at the 75 (leucine) position in the amino acid sequence of the CDC10 protein. Leucine at residue 75 in CDC10 is highly conserved across vertebrates from zebrafish and chicken to mammals (Figure 5).

### 3.4. Genetic Diversity of CDC10 Variants

For the 13 variants detected in this study, the frequencies of the two alleles and the three genotypes of each SNP in the Qinchuan cattle are listed in Table S4, as are the genetic indices (H_o_, H_e_, n_e_ and PIC). The PIC values of the g.61304737A>G, g.61303677T>G, c.225A>G, g.61224990A>G, g.61204948G>A, g.61204712A>G, and g.61204433A>T variants were low, while g.61303052G>C, g.61245443A>G, g.61209797T>G, and g.61204278C>T were moderate (high, PIC > 0.5; moderate, 0.25 < PIC < 0.5; low, PIC < 0.25) [28] in the Qinchuan cattle population (Table S4).

### 3.5. Linkage Disequilibrium Among CDC10 Variants

**Figure 6:**
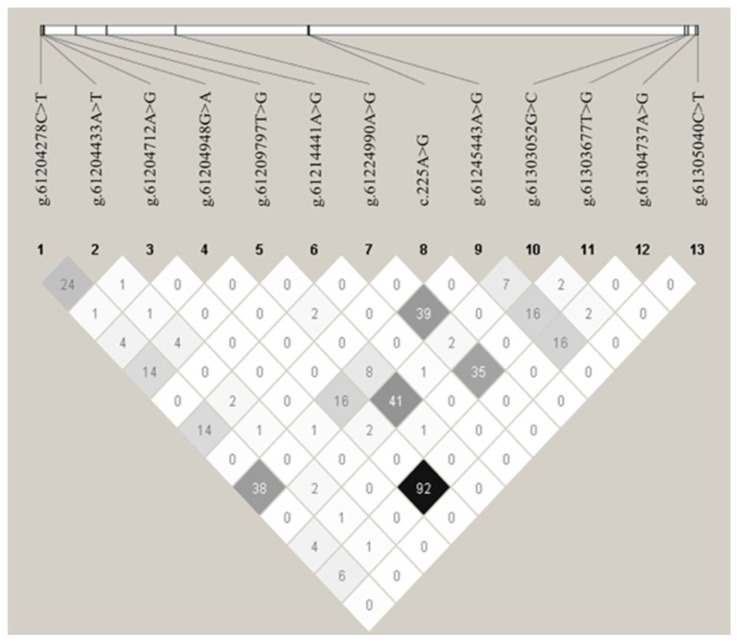
Linkage disequilibrium (LD) estimated among 13 *CDC10* SNPs in Qinchuan cattle population. “g.” indicates the position of the mutation within the *CDC10* genomic DNA, and the numbers in the squares represent the *r*^2^ values as *r*^2^ > 0.33 indicates LD. A darker color indicates a higher degree of linkage.

To determine the linkage relationships among the 13 polymorphisms, D’ and *r*^2^ were estimated for all 367 Qinchuan cattle (Table S5). The *r*^2^ values indicated that the g.61204948G>A and g.61304737A>G SNPs were in nearly complete LD in the experimental Qinchuan cattle population (*r*^2^ = 0.926) (Figure 6), as *r*^2^ > 0.33 indicates LD [29]. Thus, the LD group was collectively analyzed and designated as the single locus, denoted as LD-Q. Table S5 presents the D’ and *r*^2^ of the experimental Qinchuan cattle population.

### 3.6. Associations of CDC10 Variants with Growth-Related Traits in Qinchuan Cattle

When the number of cattle with a given genotype was less than ten, their associations and effects could not be reliably estimated. Therefore, animals with this genotype were excluded from the analysis. For g.61303677T>G, the ULD of Qinchuan cattle with the GT genotype was significantly higher than those of individuals with the TT genotype (*p* < 0.05; Table 3). For g.61303052G>C, the CCF and ULA of Qinchuan cattle with the GG genotype were highly significantly lower than those of Qinchuan cattle with the GC genotype (*p* < 0.01) and significantly lower than those of Qinchuan cattle with the CC genotype (*p* < 0.05). The BW, HW, and PWB of Qinchuan cattle with the GG genotype were significantly lower than those of Qinchuan cattle with the GC and CC genotypes (*p* < 0.05, respectively). The HH and BFT of Qinchuan cattle with the GG genotype were significantly lower than those of individuals with the GC genotype (*p* < 0.05). Meanwhile, the BFT of Qinchuan cattle with the GG genotype was highly significantly lower than that of individuals with the CC genotype (*p* < 0.01; Table 3). For the c.225A>G, the BW, CCF, ULD, and ULA of Qinchuan cattle with the GA genotype were significantly higher than those of individuals with the AA genotype (*p* < 0.05, respectively; Table 3). For g.61209797T>G, compared with the GG genotype, individuals with the GT genotype had significantly higher ULD (*p* < 0.05; Table 3). For g.61204433A>T, the ULA of Qinchuan cattle with the AT genotype were significantly higher than that of those with the TT genotype (*p* < 0.05; Table 3). Table 3 and Table S6 respectively present the mutations that remained significantly associated with, and those not significantly associated with, growth-related traits after Bonferroni correction.

To further analyze the associations of traits between haplotypes of *CDC10*, different haplotypes were constructed in the experimental population of Qinchuan cattle using the online tool SHEsis (Table S7). A haplotype with a frequency of >3% was considered a distinguishable haplotype, while haplotypes with a relative frequency of <3% were pooled into a single group. The results showed that there were no significant differences in 13 traits between *CDC10* haplotypes in Qinchuan cattle (Table S8).

### 3.7. Bioinformatics Characterization of Bovine CDC10

#### 3.7.1. Feature and Structure Prediction of the Bovine CDC10 Protein

**Figure 7:**
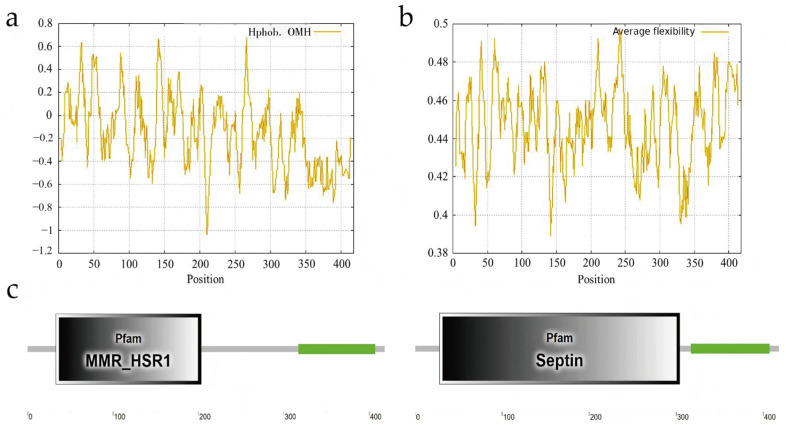
Features and structure prediction of the bovine CDC10 protein. (**a**) Hydrophobicity analysis of the CDC10 protein. (**b**) Average flexibility index of the CDC10 protein. (**c**) Predicted conserved domains of the CDC10 protein.

Hydrophobicity analysis [30] of the bovine CDC10 protein indicated that the maximum hydrophobicity value was 0.677 at 266 aa and that the minimum was −1.041 at 211 aa (Figure 7a). The maximum and minimum average flexibility index values of bovine CDC10 were 0.498 for the 242 aa position and 0.389 for the 142 aa position (Figure 7b). Then, SMART was used to predict the conserved domain. The prediction revealed a Septin-type guanine nucleotide-binding (G) domain at 28–304, a Homogeneously Staining Region at 33–203, and a low complexity region at 317–407 (Figure 7c). The silent mutation c.225A>G (75L) is located in the Septin-type guanine nucleotide-binding (G) domain and Homogeneously Staining Region of CDC10, both of which are critical for the biological function of the CDC10 protein. Therefore, the mutation should be considered more important than the other mutations found in this study.

#### 3.7.2. Amino Acid Sequence Analysis of Bovine CDC10

ProtParam was used to predict the physicochemical properties of amino acid sequences. The molecular weight and isoelectric point of bovine CDC10 were 48,757.89 Da and 8.76, respectively. The amino acid composition of the CDC10 protein showed that the highest proportion of the CDC10 protein was 11.0% for lysine and that the lowest was 0.7% for tryptophan.

**Figure 8:**
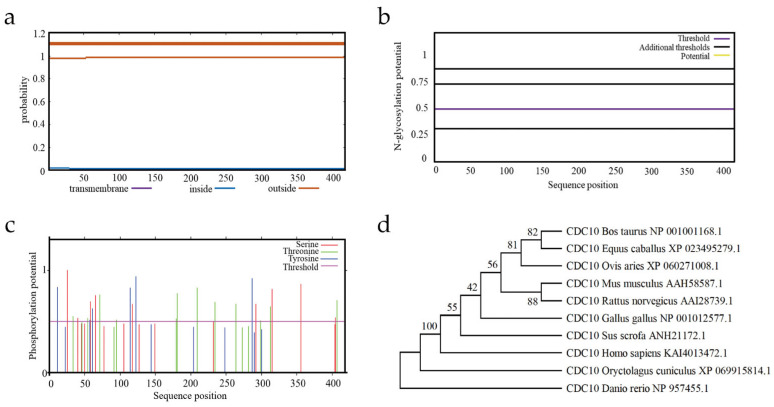
Amino acid sequence analysis of bovine CDC10 and its multiple sequence alignments. (**a**) Predicted transmembrane helices of the CDC10 protein. (**b**) Predicted N-glycosylation sites of the CDC10 protein. (**c**) Predicted phosphorylation sites of the CDC10 protein. (**d**) Phylogenetic tree based on the homologous amino acid sequence for CDC10.

Because of the protein function and its physical and chemical environment in vivo, TMHMM was used to predict the subcellular localization. The protein has no transmembrane helix position on CDC10 (Figure 8a). Furthermore, no N-glycosylation sites were identified, while 27 phosphorylation sites (comprising 12 threonines, nine serines and six tyrosines) were predicted (Figure 8b,c).

#### 3.7.3. Multiple Sequence Alignment and Phylogenetic Analysis

Amino acid sequences of CDC10 from a range of model and domesticated animals were selected to construct a phylogenetic tree. The tree was built using the Neighbor-Joining method in MEGA 12.1 software with 1000 bootstrap replications. The tree shows that CDC10 sequences from certain laboratory mammals (e.g., mouse and rat) form a distinct cluster, separate from the branches containing zebrafish and rabbit (Figure 8d).

#### 3.7.4. Predicted Effects of Variants on CDC10 mRNA Secondary Structure

**Figure 9:**
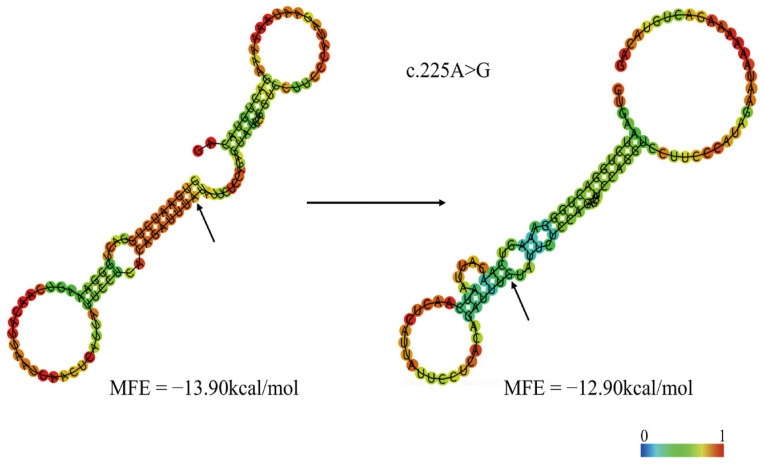
Predicted secondary structures and minimum free energies (MFE) for exon 4 of the *CDC10* gene. The structures for both the wild-type mRNA and the c.225A>G mutant are shown, colored by base-pairing probability, where darker colors indicate a higher probability of a nucleotide being unpaired. The arrow points to the c.225A>G SNP.

Using the minimum free energy (MFE) based RNAfold platform analysis, the results can show a difference in the secondary structure of the point mutation [31]. The MFE of the exonic mRNA sequence with the A allele at the c.225A>G site was −13.90 kcal/mol, while the MFE with the G allele was −12.90 kcal/mol (Figure 9). In addition, the c.225A>G mutation also caused visible changes in the mRNA secondary structure of *CDC10* (Figure 9).

## 4. Discussion

In mouse models, knockout of *CDC10* results in embryonic lethality [32]. In recent years, the *CDC10* gene has been repeatedly mapped to quantitative trait loci (QTL) affecting growth-related traits in beef cattle [12,13,14,15]. Furthermore, our previous studies found that the expression levels of the *CDC10* gene were higher in the skeletal muscle in Japanese Black beef cattle (JB) with high growth performance than in JB with low growth [16]. This suggests that the *CDC10* gene may be involved in the growth and development of bovine skeletal muscle. In this study, we initially randomly selected 3 Qinchuan cattle with low growth performance for carcass weight and 3 with high growth performance for carcass weight. Using RT-PCR and Western blot analysis to detect *CDC10* expression, the results showed that the mRNA and protein expression levels of *CDC10* was significantly higher in Qinchuan cattle with high growth performance for carcass weight compared to those with low growth performance for carcass weight. This finding is consistent with previous observations in JB cattle, where individuals with heavier carcasses also exhibited higher *CDC10* gene expression [16]. Moreover, association analysis with growth-related traits in Qinchuan cattle revealed that the g.61303052G>C SNP in the promoter region and the c.225A>G SNP in the exon region of the *CDC10* gene were significantly associated with multiple growth-related traits. This study has thereby identified potential molecular markers for Qinchuan cattle breeding. These two SNPs can serve as functional molecular markers for the future establishment of an efficient marker-assisted selection system targeting growth-related traits for this breed.

To investigate the association between the *CDC10* gene and growth-related traits in Qinchuan cattle, this study employed direct sequencing and MassARRAY technology to identify SNPs within the promoter, exons, introns, and 3′UTR regions of *CDC10*. Association analysis was then performed to identify SNPs significantly associated with growth-related traits in Qinchuan cattle. The results revealed the presence of the g.61303052G>C (ss9673029750) SNP in the promoter region of *CDC10*. This SNP was initially discovered in our previous study on Japanese Black cattle, where the C allele was shown to be significantly associated with growth-related traits [16]. In the current association analysis of the g.61303052G>C SNP with growth-related traits in Qinchuan cattle, we found that the C allele was significantly associated with multiple key growth metrics, including body weight, hip height, chest circumference, pin bone width, back fat thickness, loin muscle depth and loin muscle area. Therefore, this study confirms that the association between the g.61303052G>C SNP in the *CDC10* promoter region and growth-related traits is universal. The g.61303052G>C SNP can serve as a valuable molecular marker for subsequent breeding research.

Additionally, this study identified a c.225A>G (ss9673029758) SNP in exon 4 of the *CDC10* gene that was significantly associated with growth-related traits such as body weight, chest circumference, loin muscle depth and loin muscle area. We analyzed the amino acid change caused by this mutation and found that it resulted in the same amino acid, leucine, indicating that the c.225A>G SNP is a synonymous mutation. Nevertheless, numerous studies have shown that although synonymous mutations do not alter the amino acid sequence, they can influence mRNA expression, splicing, stability [33,34,35], secondary structure [36,37], protein translation [38] and folding, as well as protein function [39]. Research has found that mRNAs rich in optimal codons are more stable, while those rich in non-optimal codons tend to be cleared due to low translation efficiency. This provides one of the strongest pieces of evidence that synonymous mutations can affect mRNA stability by altering codon optimality [40]. Another study also indicates that the C957T synonymous mutation alters the predicted mRNA folding, leading to reduced mRNA stability and translation, and significantly impairs dopamine-induced upregulation of DRD2 expression [33]. Our analysis revealed that the minimum free energy (MFE) increased from −13.90 kcal/mol to −12.90 kcal/mol due to the c.225A>G mutation, suggesting a rise in free energy state and potential destabilization of the mRNA secondary structure. Since a lower MFE value generally indicates a more stable RNA conformation, this change implies a potential destabilizing effect on mRNA folding [36,37]. Furthermore, research has shown that the stability of mRNA secondary structure and the associated translation initiation rate play a dominant role in influencing gene expression levels. Therefore, the findings of this study indicate that the synonymous c.225A>G SNP may affect gene expression by altering the mRNA stability of the *CDC10* gene [41,42]. However, since the number of mutant samples for the c.225A>G was at the critical threshold for statistical analysis (n = 10), the results of this hypothesis are inevitably subject to certain limitations. In subsequent research, we will expand the sample size to re-examine and further analyze this locus.

In conclusion, this study demonstrated that the protein expression level of CDC10 in Qinchuan cattle with high growth performance for carcass weight was significantly higher than that in Qinchuan cattle with low growth performance for carcass weight. Association analysis revealed that the g.61303052G>C (ss9673029750) SNP in the promoter region, the c.225A>G (ss9673029758) SNP in exon 4, the g.61209797T>G (ss9673029716) SNP in intron 3, and the g.61204433A>T (ss9673029479) SNP in the 3′ UTR region of the *CDC10* gene were all significantly associated with the growth-related traits of Qinchuan cattle. Among these, the g.61303052G>C SNP was found to be significantly associated with multiple growth-related traits across various beef cattle breeds, while the c.225A>G SNP, although not causing an amino acid change, may still alter the secondary structure of *CDC10* mRNA. Therefore, the g.61303052G>C and c.225A>G SNPs will be key focuses for subsequent breeding research. In particular, how the g.61303052G>C SNP affecting the promoter activity of the *CDC10* gene will be experimentally verified through dual-luciferase reporter assays in future study. This study identified molecular markers of the *CDC10* gene associated with growth-related traits in Qinchuan cattle, providing valuable scientific insights and molecular markers for breeding improvements related to growth and development in Qinchuan cattle, as well as reliable theoretical support for the development of the beef cattle industry.

## 5. Conclusions

This study revealed that the expression of the *CDC10* gene was significantly higher at both the mRNA and protein levels in Qinchuan cattle with high growth performance for carcass weight compared to those with low growth performance for carcass weight. A promoter mutation (g.61303052G>C) in the *CDC10* gene was significantly associated with growth-related traits including BW, HH, HW, CCF, PWB, BFT and ULA, while an exonic SNP (c.225A>G) showed significant associations with BW, CCF, ULD and ULA traits. Furthermore, the c.225A>G SNP was found to alter the secondary structure of the CDC10 protein. These findings provide reliable molecular markers for improving the growth rate of Qinchuan cattle and establish a solid theoretical foundation for the beef cattle industry. In subsequent research, we will expand the sample size and conduct additional analyses on growth-related trait associated SNPs in Qinchuan cattle.

## Figures and Tables

**Table 1:** PCR primers used for the sequencing of *CDC10*.

Name	Target Region	Primer Sequence (5′–3′)	Annealing Temperature (°C)	Region (bp)
*CDC10*-P-1	promoter	F: CTCACTGCCTATAATCTTCC	54	Upstream of promoter −2391~−1616
		R: GGCAAAGGATACATGAATGTT		
*CDC10*-P-2	promoter	F: CAAGATACAAAATGAGCTGTT	55	Upstream of promoter −1734~−1141
		R: CTCATAGTTCATGTACACGTT		
*CDC10*-P-3	promoter	F: CTCACGATAATTACATAGGCAT	54	Upstream of promoter −1219~−647
		R: CTCAGTTTCCTTCACGTTC		
*CDC10*-P-4	promoter	F: ACCTCACTTTTCATTCCGAAC	56	Upstream of promoter −682~+89
		R: CCTATTCTCCAAAGCCGCCTA		
*CDC10*-1	Exon 1	F: CTGTTGCCTGCGTAAGCG	60	239 bp (119 bp 5′flanking region + 61 bp exon 1 + 59 bp intron 1)
		R: AACCCCACAACGTCCCGAG		
*CDC10*-3	Exon 3	F: CGAGTCAGTGATGTCATCTG	56	623 bp (260 bp intron 2 + 97 bp exon 3 + 266 bp intron 3)
		R: CCCACTGCTTTTCAACTTCT		
*CDC10*-4	Exon 4	F: TGTCCTCCAAGAAGTAATTGG	55	387 bp (112 bp intron 3 + 107 bp exon 4 + 168 bp intron 4)
		R: GAGACGAAAAGAACAATAACACAG		
*CDC10*-5	Exon 5	F: CACTCGTCTCCCTACATCTTACC	60	324 bp (83 bp intron 4 + 101 bp exon 5 + 140 bp intron 5)
		R: GTGGCAAAGTTGATATATGAACCC		
*CDC10*-6	Exon 6	F: CCAGGCAAGAATACTGGAAAGGG	60	376 bp (155 bp intron 5 + 135 bp exon 6 + 86 bp intron 6)
		R: CAGAGATGAGGATTACTAACACATG		
*CDC10*-7	Exon 7	F: GATGAAGAAGATGTTCTTTTGAGCC	60	658 bp (416 bp intron 6 + 118 bp exon 7 + 124 bp intron 7)
		R: CCACACACACACAGTAAAGTAGC		
*CDC10*-8	Exon 8	F: GGAACTGGAGCTAAAGACTCAG	58	494 bp (107 bp intron 7 + 93 bp exon 8 + 294 bp intron 8)
		R: CGTATCTGACTCTTTGTGACCC		
*CDC10*-9	Exon 9	F: CCGATGAAAGTGAATGCAAAAACT	60	428 bp (133 bp intron 8 + 97 bp exon 9 + 198 bp intron 9)
		R: CCTGACACAACAGGACATAAGG		
*CDC10*-10	Exon 10	F: GTGATTCCTCACCCACCCTTG	60	461 bp (186 bp intron 9 + 52 bp exon 10 + 223 bp intron 10)
		R: CTATATATCCCTAGCCACACAGCC		
*CDC10*-11	Exon 11	F: GTATCTTGCTTCCCCTTACAT	54	581 bp (153 bp intron 10 + 126 bp exon 11 + 302 bp intron 11)
		R: TATCCGACTCCTTGTGACC		
*CDC10*-12	Exon 12	F: TTCAAAAAACAGAAAGCAGAGAA	60	408 bp (129 bp intron 11 + 136 bp exon 12 + 143 bp intron 12)
		R: CGACTGAACACACAGAGACAC		
*CDC10*-13	Exon 13	F: CTGTTTGCTCATCTAGGAGTGG	60	621 bp (364 bp intron 12 + 140 bp exon 13 + 117 bp intron 13)
		R: GTGCTTGGGATGTATGATAATGGA		
*CDC10*-14	Exon 14	F: CTCATTTTGATTGTTTTCGT	58	735 bp (32 bp intron 13 + 40 bp exon 14 + 663 bp 3′flanking region)
		R: AAAATGAAGCAGTAATGAGA		
*CDC10*-3′UTR	3′UTR	F: TAGAGCTTTTTTATAACAACC	60	932 bp (3′flanking region)
		R: CTTCAGCTTCTAGTGTCAA		

Note: F, forward primer; R, reverse primer; UTR, untranslated region.

**Table 2:** MassARRAY primers for the genotyping of *CDC10*.

Polymorphism	Primers (5′–3′)	Tm (°C)
g.61305040C>T	F: ACGTTGGATGTGAGTAGGGCAAAACCACAC	56.6
	R: ACGTTGGATGGTTTGTGGGTGAAAAGCCTG	
	E: GAGAGGGAGTGGCCCGT	
g.61304737A>G	F: ACGTTGGATGCACATCTATTAAGAGATCTGC	45.1
	R: ACGTTGGATGAAAGAAGAAAGTCTCCCATC	
	E: ATCTGCTCAAAGAAACATAT	
g.61303677T>G	F: ACGTTGGATGACTATACAATGAAGACAGCG	45.6
	R: ACGTTGGATGAGTAGCCTCCTTTTGCTCAG	
	E: GTTTTATGAAAGGTACAAAAGAT	
g.61303052G>C	F: ACGTTGGATGTAGCCGCCATGTTAACATTG	48.9
	R: ACGTTGGATGGAAGAAACACGCACAGAAGC	
	E: ACTATCCATGACCCCG	
g.61245443A>G	F: ACGTTGGATGAAGCTTGATAAGTTGACTG	45.7
	R: ACGTTGGATGTGTTGACTTTCCCAGTCCAG	
	E: TTGATAAGTTGACTGAGTTTT	
c.225A>G (75L)	F: ACGTTGGATGTCTGGACTGGGAAAGTCAAC	47.9
	R: ACGTTGGATGTATGGGAAGGACCTGGATAC	
	E: CCTGGATACTCTGGAGAATA	
g.61224990A>G	F: ACGTTGGATGGCCAACAGTTTAAGAAACAGG	46.1
	R: ACGTTGGATGGGAGGAACACACTGTAACAC	
	E: GGGGAAATTCTTAGGATTCTAATACT	
g.61214441A>G	F: ACGTTGGATGCCTGCCATGAATGGCATTAT	46.7
	R: ACGTTGGATGTCTATTGAGTAGCCAAGAGG	
	E: CATGAATGGCATTATTTATTTAGTAA	
g.61209797T>G	F: ACGTTGGATGGATAGATAATGTAACCGCCC	45.7
	R: ACGTTGGATGGTTCGACTGAACACACAGAG	
	E: GAACACACAGAGACACATTA	
g.61204948G>A	F: ACGTTGGATGAGGTTCCATTCAATGCAGCG	51
	R: ACGTTGGATGGGAAGTGTCAAAAAATAGGG	
	E: GGGTCAACTGTGTTAGACTAAATTA	
g.61611958A>G	F: ACGTTGGATGAGCCAGAATCTCATTACTGC	50.7
	R: ACGTTGGATGGTTTCCTTGTTGGTTCCTATC	
	E: TCTATTTTTTAGCAGGGCCAATA	
g.61204712A>G	F: ACGTTGGATGCACCATTCTGCATTTAGCTG	45.1
	R: ACGTTGGATGCCCACACTCAATTACATCAC	
	E: GTATTCATATATTGCATTTCTGTA	
g.61204278C>T	F: ACGTTGGATGGAATTTGCCTTCTCCATGTT	52.8
	R: ACGTTGGATGGGAGACAGTAGTAAGTAGAC	
	E: TGCCTTCTCCATGTTTGTAACT	

Note: F: forward primer sequence; R: reverse primer sequence; E: extended primer sequence; Tm: melting temperature.

**Table 3:** Association between the SNPs of the *CDC10* gene and growth-related traits in Qinchuan cattle.

SNP	Genotype	BW	BL	WH	HH	RL	HW	CD	CCF	PWB	BFT	ULD	ULA	IMF
	(No.)	(kg)	(cm)	(cm)	(cm)	(cm)	(cm)	(cm)	(cm)	(cm)	(cm)	(cm)	(cm^2^)	(%)
g.61303677T>G	TT (328)	337.77 ± 5.61	134.16 ± 0.74	123.92 ± 0.42	121.29 ± 0.49	41.93 ± 0.25	38.63 ± 0.32	58.84 ± 0.37	162.56 ± 0.97	18.74 ± 0.17	0.89 ± 0.02	4.48 ± 0.07 ^a^	46.23 ± 0.91	7.48 ± 0.06
	GT (39)	360.50 ± 18.3	135.67 ± 2.07	123.74 ± 1.29	121.76 ± 1.48	42.59 ± 0.78	39.44 ± 0.97	59.73 ± 1.10	166.72 ± 3.22	18.99 ± 0.60	0.95 ± 0.07	4.96 ± 0.23 ^b^	50.38 ± 2.53	7.25 ± 0.17
g.61303052G>C	GG (23)	281.38 ± 17.27 ^a^	128.17 ± 2.48	121.46 ± 1.36	117.02 ± 1.47 ^a^	40.78 ± 0.62	35.48 ± 0.89 ^a^	56.87 ± 1.22	152.13 ± 3.04 ^Aa^	17.04 ± 0.54 ^a^	0.69 ± 0.03 ^Aa^	3.90 ± 0.27	37.2 ± 2.42 ^Aa^	7.58 ± 0.18
	GC (161)	346.77 ± 8.08 ^b^	135.15 ± 1.09	124.59 ± 0.63	122.38 ± 0.72 ^b^	42.22 ± 0.38	39.11 ± 0.48 ^b^	59.24 ± 0.55	164.16 ± 1.39 ^B^	18.99 ± 0.25 ^b^	0.89 ± 0.02 ^b^	4.55 ± 0.10	48.08 ± 1.33 ^B^	7.47 ± 0.08
	CC (183)	341.78 ± 7.69 ^b^	134.36 ± 0.96	123.60 ± 0.56	120.96 ± 0.64	41.96 ± 0.33	38.78 ± 0.43 ^b^	58.93 ± 0.49	163.35 ± 1.33 ^b^	18.79 ± 0.24 ^b^	0.92 ± 0.03 ^B^	4.58 ± 0.10	46.63 ± 1.20 ^b^	7.43 ± 0.08
c.225A>G (75L)	AA (357)	338.35 ± 5.47 ^a^	134.18 ± 0.71	123.84 ± 0.41	121.27 ± 0.47	41.99 ± 0.24	38.66 ± 0.31	58.84 ± 0.36	162.61 ± 0.95 ^a^	18.74 ± 0.17	0.89 ± 0.02	4.50 ± 0.07 ^a^	46.33 ± 0.87 ^a^	7.46 ± 0.05
	GA (10)	405.82 ± 21.8 ^b^	139.20 ± 3.71	125.95 ± 2.18	123.90 ± 2.25	42.2 ± 1.58	40.90 ± 1.36	62.45 ± 1.71	176.80 ± 2.63 ^b^	19.70 ± 0.79	0.98 ± 0.11	5.49 ± 0.15 ^b^	58.99 ± 3.01 ^b^	7.32 ± 0.30
g.61209797T>G	TT (233)	342.39 ± 6.94	134.45 ± 0.87	123.69 ± 0.51	121.22 ± 0.58	41.92 ± 0.29	38.66 ± 0.38	59.07 ± 0.44	163.37 ± 1.20	18.69 ± 0.21	0.92 ± 0.02	4.53 ± 0.08	47.01 ± 1.10	7.50 ± 0.06
	GT (120)	338.55 ± 9.07	134.04 ± 1.21	124.30 ± 0.69	121.69 ± 0.82	42.15 ± 0.42	38.92 ± 0.55	58.85 ± 0.63	162.93 ± 1.59	19.01 ± 0.29	0.87 ± 0.03	4.61 ± 0.12 ^a^	47.17 ± 1.46	7.34 ± 0.10
	GG (14)	317.45 ± 24.29	134.50 ± 4.47	124.07 ± 2.39	120.29 ± 2.52	42.00 ± 1.55	37.93 ± 1.27	57.46 ± 1.62	157.57 ± 3.84	18.07 ± 0.77	0.71 ± 0.05	3.72 ± 0.43 ^b^	36.65 ± 2.22	7.64 ± 0.21
g.61204433A>T	AA (13)	352.75 ± 32.53	135.85 ± 4.63	124.27 ± 2.04	121.96 ± 2.56	42.23 ± 1.26	38.62 ± 1.52	60.96 ± 2.27	164.62 ± 5.23	19.00 ± 0.82	0.81 ± 0.05	4.56 ± 0.30	45.64 ± 4.24	7.34 ± 0.28
	AT (94)	347.04 ± 11.69	134.51 ± 1.43	123.55 ± 0.86	121.29 ± 0.92	41.77 ± 0.45	38.81 ± 0.64	59.05 ± 0.76	164.14 ± 2.00	18.85 ± 0.36	0.93 ± 0.04	4.61 ± 0.12	50.47 ± 1.76 ^a^	7.63 ± 0.08
	TT (260)	337.08 ± 6.12	134.17 ± 0.81	124.01 ± 0.47	121.32 ± 0.55	42.07 ± 0.28	38.69 ± 0.36	58.79 ± 0.40	162.51 ± 1.07	18.73 ± 0.20	0.88 ± 0.02	4.49 ± 0.08	45.35 ± 1.00 ^b^	7.40 ± 0.07

Note: Values are shown as the means ± standard error. Values with different superscript letters within the same column differ significantly: a,b (*p* < 0.05); A,B (*p* < 0.01) after Bonferroni correction. BW, Body weight; BL, Body length; WH, Wither height; HH, Hip height; RL, Rump length; HW, Hip width; CD, Chest depth; CCF, Chest circumference; PWB, Pin bone width; BFT, Ultrasound back fat thickness; ULD, Ultrasound loin muscle depth; ULA, Ultrasound loin muscle area; IMF, Ultrasound intramuscular fat.

## Data Availability

The original contributions presented in this study are included in the article/Supplementary Materials. Further inquiries can be directed to the corresponding author.

## Supplementary Materials

The following supporting information can be downloaded at: https://www.mdpi.com/article/10.3390/ani16030447/s1, Table S1. Bioinformatics tools and resources used in the analysis. Table S2: Studied traits and their abbreviations; Table S3: Pairwise correlations among the 13 studied traits; Table S4: Genotypic frequencies, allelic frequencies, and diversity parameters of 13 variants in *CDC10* of Qinchuan cattle population; Table S5: Linkage disequilibrium as measured by D’ and *r*^2^ among variants in the *CDC10*; Table S6: Association between the SNPs of the *CDC10* gene and growth-related traits in Qinchuan cattle; Table S7: Main haplotypes and their frequencies of *CDC10* in Qinchuan cattle. Table S8. Differences of 13 traits between haplotypes of *CDC10* in Qinchuan cattle.
