# Supplementary material for: Association of Variants in CDC10 (Septin 7) Gene with Growth-Related Traits in Qinchuan Cattle
Source: Animals (Basel). 2026 Feb 1;16(3):447. doi: 10.3390/ani16030447 (PMC12897450; doi:10.3390/ani16030447)
Supplement: Supplementary file 1 [file animals-16-00447-s001.zip › animals-4076098-supplementary.pdf]

Table S1. Bioinformatics tools and resources used in the analysis.

Tool Name	Purpose	Website / Resource
ProtParam	Analysis of physicochemical properties	https://web.expasy.org/protparam/
TMHMM	Identification of potential transmembrane regions	http://www.cbs.dtu.dk/services/TMHMM-2.0
SMART	Examination of conserved domains	http://smart.embl-heidelberg.de/
NetNGlyc	Prediction of N-glycosylation sites	https://services.healthtech.dtu.dk/services/NetNGlyc-1.0/
NetPhos	Prediction of phosphorylation sites	https://services.healthtech.dtu.dk/service.php?NetPhos-3.1
ProtScale	Assessment of hydrophilicity and average flexibility index	http://web.expasy.org/protscale/
RNAfold	Prediction of *CDC10* mRNA secondary structure	http://rna.tbi.univie.ac.at/cgi-bin/RNAWebSuite/RNAfold.cgi
UniProt Align	Multiple sequence alignment	https://www.uniprot.org/align
MEGA-X	Phylogenetic analysis	https://www.megasoftware.net/docs

Table S2. Studied traits and their abbreviations.

Traits	Abbreviation
Body weight	BW
Body length	BL
Wither height	WH
Hip height	HH
Rump length	RL
Hip width	HW
Chest depth	CD
Chest circumference	CCF
Pin bone width	PWB
Ultrasound back fat thickness	BFT
Ultrasound loin muscle depth	ULD
Ultrasound loin muscle area	ULA
Ultrasound Intramuscular fat	IMF

Note: This table lists the full names and corresponding abbreviations for the 13 traits studied in the Qinchuan cattle population.

Table S3. Pairwise correlations among the 13 studied traits.

Traits	BW	BL	WH	HH	RL	HW	CD	CCF	PWB	BFT	ULD	ULA	IMF
BW	1	0.898**	0.816**	0.859**	0.739**	0.852**	0.845**	0.974**	0.778**	0.655**	0.488**	0.514**	0.175**
BL	0.898**	1	0.785**	0.803**	0.715**	0.764**	0.778**	0.800**	0.696**	0.488**	0.404**	0.401**	0.195**
WH	0.816**	0.785**	1	0.903**	0.722**	0.755**	0.783**	0.795**	0.722**	0.426**	0.398**	0.407**	0.183**
HH	0.859**	0.803**	0.903**	1	0.779**	0.821**	0.832**	0.839**	0.751**	0.485**	0.459**	0.462**	0.201**
RL	0.739**	0.715**	0.722**	0.779**	1	0.796**	0.728**	0.715**	0.627**	0.462**	0.368**	0.348**	0.112*
HW	0.852**	0.764**	0.755**	0.821**	0.796**	1	0.805**	0.851**	0.768**	0.587**	0.421**	0.422**	0.139**
CD	0.845**	0.778**	0.783**	0.832**	0.728**	0.805**	1	0.829**	0.697**	0.514**	0.431**	0.457**	0.143**
CCF	0.974**	0.800**	0.795**	0.839**	0.715**	0.851**	0.829**	1	0.791**	0.668**	0.475**	0.520**	0.191**
PWB	0.778**	0.696**	0.722**	0.751**	0.627**	0.768**	0.697**	0.791**	1	0.530**	0.379**	0.463**	0.233**
BFT	0.655**	0.488**	0.426**	0.485**	0.462**	0.587**	0.514**	0.668**	0.530**	1	0.392**	0.505**	0.115*
ULD	0.488**	0.404**	0.398**	0.459**	0.368**	0.421**	0.431**	0.475**	0.379**	0.392**	1	0.524**	0.161**
ULA	0.514**	0.401**	0.407**	0.462**	0.348**	0.422**	0.457**	0.520**	0.463**	0.505**	0.524**	1	0.234**
IMF	0.175**	0.195**	0.183**	0.201**	0.112*	0.139**	0.143**	0.191**	0.233**	0.115*	0.161**	0.234**	1

Note: *$p < 0.05$; **$p < 0.01$.

Table S4. Genotypic frequencies, allelic frequencies and diversity parameters of 13 variants in *CDC10* of Qinchuan cattle population.

Variants	Genotype frequency			Allele frequency		Diversity parameter				
						H_o	H_e	n_e	PIC	χ^2 (HWE[2])
g.61305040C>T	CC	CT	TT	C	T					
	0.989	0.011	0.000	0.995	0.005	0.989	0.011	1.011	0.010	0.011
g.61304737A>G	AA	GA	GG	A	G					
	0.891	0.109	0.000	0.946	0.054	0.897	0.103	1.115	0.097	1.219
g.61303677T>G	TT	GT	GG	T	G					
	0.894	0.106	0.000	0.947	0.053	0.899	0.101	1.112	0.095	1.156
g.61303052G>C	GG	GC	CC	G	C					
	0.063	0.439	0.499	0.282	0.718	0.595	0.405	1.681	0.323	2.545
g.61245443A>G	AA	AG	GG	A	G					
	0.063	0.392	0.545	0.259	0.741	0.616	0.384	1.623	0.310	0.187
c.225A>G (75L)	AA	GA	GG	A	G					
	0.973	0.027	0.000	0.986	0.014	0.973	0.027	1.028	0.027	0.070
g.61224990A>G	AA	AG	GG	A	G					
	0.766	0.213	0.022	0.872	0.128	0.777	0.223	1.288	0.198	0.858
g.61214441A>G	AA	AG	GG	A	G					
	0.992	0.008	0.000	0.996	0.004	0.992	0.008	1.008	0.008	0.006
g.61209797T>G	TT	GT	GG	T	G					
	0.635	0.327	0.038	0.798	0.202	0.678	0.322	1.475	0.270	0.089
g.61204948G>A	GG	GA	AA	G	A					
	0.886	0.112	0.003	0.941	0.059	0.890	0.110	1.124	0.105	0.060
g.61204712A>G	AA	AG	GG	A	G					
	0.861	0.128	0.011	0.925	0.075	0.861	0.139	1.161	0.129	2.133
g.61204433A>T	AA	AT	TT	A	T					
	0.035	0.256	0.708	0.163	0.837	0.726	0.274	1.376	0.236	1.483
g.61204278C>T	CC	CT	TT	C	T					
	0.210	0.471	0.319	0.446	0.554	0.506	0.494	1.977	0.372	0.773

Note: Ho: observed heterozygosity, He: expected heterozygosity, ne: effective allele numbers, PIC: polymorphism information content, HWE: Hardy-Weinberg equilibrium. The classification was conducted according to the PIC values (PIC value < 0.25, low polymorphism; $0.25 <$ PIC value < 0.5, moderate polymorphism; and PIC value > 0.5, high polymorphism). No Hardy-Weinberg departure was detected from the obtained genotype frequencies.

Table S5. Linkage disequilibrium as measured by D' and r^2 among variants in the *CDC10*.

SNPs	g.61204278C>T	g.61204433A>T	g.61204712A>G	g.61204948G>A	g.61209797T>G	g.61214441A>G	g.61224990A>G	c.225A>G	g.61245443A>G	g.61303052G>C	g.61303677T>G	g.61304737A>G
g.61204433A>T	D'=1.000/ r^2=0.243											
g.61204712A>G	D'=0.522/ r^2=0.018	D'=0.968/ r^2=0.015										
g.61204948G>A	D'=0.785/ r^2=0.048	D'=1.000/ r^2=0.012	D'=0.041/ r^2=0.001									
g.61209797T>G	D'=0.841/ r^2=0.144	D'=1.000/ r^2=0.049	D'=0.291/ r^2=0.002	D'=0.305/ r^2=0.001								
g.61214441A>G	D'=1.000/ r^2=0.003	D'=0.009/ r^2=0.000	D'=0.217/ r^2=0.002	D'=1.000/ r^2=0.000	D'=0.528/ r^2=0.005							
g.61224990A>G	D'=0.885/ r^2=0.143	D'=1.000/ r^2=0.029	D'=0.291/ r^2=0.001	D'=0.019/ r^2=0.000	D'=0.823/ r^2=0.025	D'=1.000/ r^2=0.001						
c.225A>G	D'=0.644/ r^2=0.007	D'=0.436/ r^2=0.013	D'=1.000/ r^2=0.001	D'=1.000/ r^2=0.001	D'=0.705/ r^2=0.002	D'=1.000/ r^2=0.000	D'=0.060/ r^2=0.000					
g.61245443A>G	D'=0.941/ r^2=0.385	D'=0.032/ r^2=0.000	D'=0.732/ r^2=0.015	D'=0.960/ r^2=0.164	D'=1.000/ r^2=0.088	D'=1.000/ r^2=0.001	D'=0.967/ r^2=0.393	D'=0.725/ r^2=0.003				
g.61303052G>C	D'=0.078/ r^2=0.002	D'=0.219/ r^2=0.024	D'=0.403/ r^2=0.005	D'=1.000/ r^2=0.024	D'=0.807/ r^2=0.418	D'=1.000/ r^2=0.010	D'=0.660/ r^2=0.025	D'=0.998/ r^2=0.005	D'=0.757/ r^2=0.079			
g.61303677T>G	D'=0.841/ r^2=0.049	D'=1.000/ r^2=0.011	D'=0.658/ r^2=0.002	D'=0.284/ r^2=0.000	D'=1.000/ r^2=0.014	D'=1.000/ r^2=0.000	D'=0.968/ r^2=0.358	D'=0.127/ r^2=0.000	D'=1.000/ r^2=0.161	D'=1.000/ r^2=0.022		
g.61304737A>G	D'=0.920/ r^2=0.061	D'=1.000/ r^2=0.011	D'=0.781/ r^2=0.003	D'=1.000/ r^2=0.926	D'=0.585/ r^2=0.005	D'=1.000/ r^2=0.000	D'=0.377/ r^2=0.001	D'=1.000/ r^2=0.001	D'=1.000/ r^2=0.165	D'=1.000/ r^2=0.023	D'=0.668/ r^2=0.001	
g.61305040C>T	D'=1.000/ r^2=0.007	D'=0.591/ r^2=0.000	D'=1.000/ r^2=0.000	D'=0.151/ r^2=0.002	D'=1.000/ r^2=0.001	D'=1.000/ r^2=0.000	D'=0.057/ r^2=0.000	D'=1.000/ r^2=0.000	D'=0.107/ r^2=0.000	D'=0.380/ r^2=0.000	D'=0.162/ r^2=0.003	D'=0.159/ r^2=0.002

Table S6. Association between the SNPs of the *CDC10* gene and growth-related traits in Qinchuan cattle.

SNPs	Genotype (No.)	BW (kg)	BL (cm)	WH (cm)	HH (cm)	RL (cm)	HW (cm)	CD (cm)	CCF (cm)	PWB (cm)	BFT (cm)	ULD (cm)	ULA (cm²)	IMF (%)
g.61304737A>G	AA (327)	339.76 ± 5.61	134.41 ± 0.74	123.98 ± 0.42	121.33 ± 0.48	42.04 ± 0.25	38.73 ± 0.32	58.99 ± 0.37	162.94 ± 0.97	18.82 ± 0.18	0.88 ± 0.02	4.53 ± 0.07	46.70 ± 0.92	7.43 ± 0.06
	GA (40)	343.68 ± 18.62	133.58 ± 2.24	123.28 ± 1.36	121.39 ± 1.64	41.65 ± 0.79	38.60 ± 1.02	58.46 ± 1.20	163.50 ± 3.30	18.39 ± 0.55	0.97 ± 0.06	4.52 ± 0.21	46.41 ± 2.45	7.61 ± 0.14
g.61245443A>G	AA (23)	344.32 ± 24.03	132.87 ± 2.76	122.20 ± 1.59	120.57 ± 1.99	41.83 ± 0.71	38.87 ± 1.20	58.61 ± 1.40	164.35 ± 4.25	18.72 ± 0.83	0.94 ± 0.09	4.32 ± 0.34	44.68 ± 3.70	7.44 ± 0.18
	AG (144)	339.61 ± 8.53	134.44 ± 1.10	123.70 ± 0.64	121.24 ± 0.74	42.12 ± 0.40	38.97 ± 0.49	58.81 ± 0.55	162.85 ± 1.50	18.72 ± 0.26	0.89 ± 0.03	4.64 ± 0.10	46.08 ± 1.30	7.46 ± 0.09
	GG (200)	340.12 ± 7.27	134.40 ± 0.96	124.24 ± 0.55	121.50 ± 0.63	41.94 ± 0.31	38.52 ± 0.42	59.07 ± 0.48	162.96 ± 1.24	18.81 ± 0.22	0.89 ± 0.02	4.47 ± 0.09	47.33 ± 1.19	7.45 ± 0.07
g.61224990A>G	AA (281)	340.18 ± 6.15	134.21 ± 0.82	124.07 ± 0.46	121.44 ± 0.53	41.87 ± 0.27	38.57 ± 0.35	58.93 ± 0.40	163.05 ± 1.06	18.83 ± 0.19	0.89 ± 0.02	4.48 ± 0.08	47.00 ± 0.98	7.51 ± 0.06
	AG (78)	338.66 ± 11.47	134.67 ± 1.37	123.54 ± 0.86	121.21 ± 0.97	42.42 ± 0.52	39.23 ± 0.70	58.88 ± 0.72	162.56 ± 2.05	18.58 ± 0.38	0.88 ± 0.04	4.62 ± 0.14	45.27 ± 1.81	7.28 ± 0.14
g.61204948G>A	GG (325)	339.43 ± 5.64	134.37 ± 0.74	123.96 ± 0.42	121.30 ± 0.48	42.02 ± 0.25	38.70 ± 0.32	58.99 ± 0.37	162.88 ± 0.97	18.80± 0.18	0.88 ± 0.02	4.52 ± 0.07	46.77 ± 0.92	7.43 ± 0.06
	GA (41)	346.89 ± 18.22	133.83 ± 2.20	123.61 ± 1.33	121.74 ± 1.60	41.85 ± 0.78	38.90 ± 1.01	58.54 ± 1.18	164.17 ± 3.23	18.55 ± 0.55	0.96 ± 0.06	4.57 ± 0.20	45.88 ± 2.42	7.62 ± 0.14
g.61204712A>G	AA (316)	339.39 ± 5.84	134.08 ± 0.76	123.90 ± 0.43	121.31 ± 0.50	42.03 ± 0.26	38.63 ± 0.34	59.03 ± 0.38	162.92 ± 1.01	18.68 ± 0.18	0.89 ± 0.02	4.54 ± 0.08	46.52 ± 0.90	7.47 ± 0.06
	GA (47)	341.09 ± 14.81	135.41 ± 1.83	123.74 ± 1.16	121.38 ± 1.28	41.66 ± 0.66	39.00 ± 0.76	58.16 ± 1.02	162.68 ± 2.64	19.28 ± 0.53	0.95 ± 0.06	4.38 ± 0.16	48.00 ± 2.84	7.31 ± 0.16
g.61204278C>T	CC (77)	356.04 ± 13.10	135.95 ± 1.60	124.04 ± 0.88	122.39 ± 1.04	42.14 ± 0.52	39.35 ± 0.67	59.60 ± 0.74	165.44 ± 2.21	18.80 ± 0.41	0.92 ± 0.04	4.65 ± 0.16	47.72 ± 2.01	7.55 ± 0.08

CT (173)	334.43 ± 7.85	133.62 ± 1.03	123.30 ± 0.59	120.48 ± 0.66	41.76 ± 0.36	38.38 ± 0.46	58.68 ± 0.54	161.97 ± 1.36	18.55 ± 0.24	0.90 ± 0.02	4.53 ± 0.10	46.05 ± 1.18	7.51 ± 0.08
TT (117)	338.26 ± 8.72	134.29 ± 1.18	124.70 ± 0.70	121.91 ± 0.84	42.27 ± 0.39	38.79 ± 0.53	58.88 ± 0.58	162.91 ± 1.54	19.08 ± 0.29	0.87 ± 0.03	4.44 ± 0.12	46.90 ± 1.57	7.30 ± 0.11

Note: Values are shown as the means ± standard error. BW, Body weight; BL, Body length; WH, Wither height; HH, Hip height; RL, Rump length; HW, Hip width; CD, Chest depth; CCF, Chest circumference; PWB, Pin bone width; BFT, Ultrasound Back fat thickness; ULD, Ultrasound loin muscle depth; ULA, Ultrasound loin muscle area; IMF, Ultrasound Intramuscular fat.

Table S7. Main haplotypes and their frequencies of *CDC10* in Qinchuan cattle.

Haplotype	g.61305040C>T	g.61304737A>G	g.61303677T>G	g.61303052G>C	g.61245443A>G	c.225 A>G	g.61224990A>G	g.61214441A>G	g.61209797T>G	g.61204948G>A	g.61204712A>G	g.61204433A>T	g.61204278C>T	Frequency
H1(212)	C	A	T	C	G	A	A	A	T	G	A	T	T	0.289
H2(118)	C	A	T	G	G	A	A	A	G	G	A	T	T	0.161
H3(51)	C	A	T	G	G	A	A	A	T	G	A	A	C	0.070
H4(44)	C	A	T	C	A	A	G	A	T	G	A	T	C	0.060
H5(44)	C	A	T	C	G	A	A	A	T	G	G	T	T	0.060
H6(37)	C	G	T	C	A	A	A	A	T	A	A	T	C	0.050
H7(36)	C	A	G	C	A	A	G	A	T	G	A	T	C	0.049
H8(35)	C	A	T	C	G	A	A	A	T	G	A	A	C	0.048
H9(30)	C	A	T	C	A	A	A	A	T	G	A	T	C	0.041
H10(25)	C	A	T	C	A	A	A	A	T	G	A	A	C	0.034

Note: A haplotype with a frequency of >3% was considered as a distinguishable haplotype.

Table S8. Differences of 13 traits between haplotypes of *CDC10* in Qinchuan cattle.

Haplotype	Number	Frequency	BW (kg)	BL (cm)	WH (cm)	HH (cm)	RL (cm)	HW (cm)	CD (cm)	CCF (cm)	PWB (cm)	BFT (cm)	ULD (cm)	ULA (cm^2)	IMF (%)
H1-H1	36	0.10	343.31 ± 16.27	135.19 ± 2.28	123.96 ± 1.27	121.90 ± 1.47	41.78 ± 0.70	38.47 ± 1.02	59.35 ± 1.00	163.42 ± 2.79	18.83 ± 0.52	0.92 ± 0.06	4.66 ± 0.21	47.36 ± 2.52	7.59 ± 0.16
H1-H2	37	0.10	324.53 ± 13.63	131.65 ± 1.95	125.28 ± 1.25	122.27 ± 1.59	42.27 ± 0.73	38.54 ± 0.97	59.14 ± 1.07	161.65 ± 2.56	19.16 ± 0.46	0.87 ± 0.05	4.55 ± 0.19	48.74 ± 2.86	7.10 ± 0.20
H1-H3	11	0.03	373.55 ± 31.62	136.86 ± 3.97	123.86 ± 2.88	122.36 ± 2.79	42.45 ± 1.23	40.09 ± 1.89	63.18 ± 2.14	169.64 ± 5.61	18.73 ± 0.84	0.96 ± 0.11	4.00 ± 0.18	47.97 ± 5.79	7.79 ± 0.12
H1-H8	12	0.03	284.08 ± 29.68	127.46 ± 4.10	118.33 ± 2.61	116.25 ± 2.76	39.5 ± 1.40	34.75 ± 2.15	55.63 ± 3.11	152.58 ± 5.03	16.83 ± 1.09	0.87 ± 0.08	4.33 ± 0.42	46.60 ± 3.57	7.54 ± 0.20

Note: Values are shown as the means ± standard error. BW, Body weight; BL, Body length; WH, Wither height; HH, Hip height; RL, Rump length; HW, Hip width; CD, Chest depth; CCF, Chest circumference; PWB, Pin bone width; BFT, Ultrasound Back fat thickness; ULD, Ultrasound loin muscle depth; ULA, Ultrasound loin muscle area; IMF, Ultrasound Intramuscular fat.
